# Secukinumab Loss of Efficacy Is Perfectly Counteracted by the Introduction of Combination Therapy (Rescue Therapy): Data from a Multicenter Real-Life Study in a Cohort of Italian Psoriatic Patients That Avoided Secukinumab Switching

**DOI:** 10.3390/ph15010095

**Published:** 2022-01-14

**Authors:** Giovanni Damiani, Giulia Odorici, Alessia Pacifico, Aldo Morrone, Rosalynn R. Z. Conic, Tima Davidson, Abdulla Watad, Paolo D. M. Pigatto, Delia Colombo, Piergiorgio Malagoli, Marco Fiore

**Affiliations:** 1 Clinical Dermatology, IRCCS Istituto Ortopedico Galeazzi, 20161 Milan, Italy; dr.giovanni.damiani@gmail.com (G.D.); paolo.pigatto@unimi.it (P.D.M.P.); 2 Department of Biomedical, Surgical and Dental Sciences, University of Milan, 20122 Milan, Italy; 3 PhD Degree Program in Pharmacological Sciences, Department of Pharmaceutical and Pharmacological Sciences, University of Padua, 35131 Padua, Italy; 4 Department of Dermatology, University of Ferrara, 44124 Ferrara, Italy; giulai87@hotmail.com; 5 Clinical Dermatology Department, San Gallicano Dermatological Institute, IRCCS, 00144 Rome, Italy; alessia.pacifico@gmail.com (A.P.); aldomorrone54@gmail.com (A.M.); 6 Department of Preventive Medicine, Maryland University, Baltimore, MD 21201, USA; ruzica.conic@gmail.com; 7 Department of Nuclear Medicine, Chaim Sheba Medical Center, Tel Hashomer 52621, Israel; tima.davidson@sheba.health.gov.il; 8 Sackler Faculty of Medicine, Tel Aviv University, Tel Aviv 6997801, Israel; watad.abdulla@gmail.com; 9 Rheumatology Unit, Department of Medicine B, Zabludowicz Center for Autoimmune Diseases, Sheba Medical Center, Ramat Gan 5265601, Israel; 10 Leeds Institute of Rheumatic and Musculoskeletal Medicine (LIRMM), University of Leeds, Leeds LS7 4SA, UK; 11 Department of Pharmacology, University of Milan, 20133 Milan, Italy; studio.deliacolombo@gmail.com; 12 Dermatology Unit, Azienda Ospedaliera San Donato Milanese, 20097 Milan, Italy; dermapier@gmail.com; 13 Department of Women, Child and General and Specialized Surgery, University of Campania “Luigi Vanvitelli”, 80138 Naples, Italy

**Keywords:** secukinumab, combination therapy, biologic multifailure, psoriasis, IL-17 inhibitors

## Abstract

Since psoriasis (PsO) is a chronic inflammatory disease, patients may experience a drug failure also with very effective drugs (i.e., secukinumab) and, consequently, dermatologists have two therapeutic options: switching or perform a combination therapy (rescue therapy) to save the drug that had decreased its efficacy. At the moment no studies focused on combination/rescue therapy of secukinumab, so we performed a 52-weeks multicenter retrospective observational study that involved 40 subjects with plaque psoriasis that experienced a secondary failure and were treated with combination therapy (ciclosporin (*n* = 11), MTX (*n* = 15), NB-UVB (*n* = 7) and apremilast (*n* = 7)). After 16 weeks of rescue/combination therapy, PASI and a DLQI varied respectively from 8 [7.0–9.0] and 13 [12.0–15.0], to 3 [2.8–4.0] and 3 [2.0–3.3]), suggesting a significant improvement of daily functionality and quality of life. Results were maintained at 52 weeks. No side effects were experienced during the study. Secukinumab remains a safety and effective drug for PsO patients also in the IL-23 and JAK inhibitors era. The rescue therapy is a valid therapeutic option in case of secukinumab secondary failure.

## 1. Introduction

Due to the chronic nature of psoriasis [1,2,3], the affected patients may experience several comorbidities (i.e., respiratory [4,5,6,7,8], cardiovascular [9,10,11,12,13] or gastrointestinal ones [14,15,16,17]) and therapy failures [18,19,20,21,22,23,24,25]. Thus, therapeutic strategies play a pivotal in limiting the psoriasis detrimental progression and the quality of life worsening. At the moment psoriasis guidelines recommend to use in label therapies, based on evidence derived from real-life studies and/or clinical trials. These clinical suggestions remain totally useless in predicting drug-response of a particular patient. In fact, target therapies were introduced to antagonize a particular pro-inflammatory cytokine (i.e., IL-17 or TNF-alpha) and did not account for the biological fingerprint of the single patient (precision medicine) [26,27,28,29]. Since no biomarkers or even predicting algorithms are validated, dermatologists orient their prescriptions only with clinical experience and the available dermato-epidemiology [30,31,32].

Nowadays, several anti-psoriatic systemic therapies are marketed, but, at the same time, also multi-failure (>2 biologics failed) patients are not rare [21]. Due to this intricated scenario, the rationale for switching, instead using a combined therapy, is a matter of debate. To date, different reasons (i.e., pharmaco-economically (originator to biosimilar [33]), insurance [34], COVID-19 pandemic [21]) lead to several drug switchings in a single patient. Interestingly, data toward combination therapies in psoriatic patients remained scarce [35,36,37,38], so we performed a retrospective observational study including all patients treated secukinumab, who underwent a secondary loss of efficacy and started another concomitant systemic (rescue therapy) treatment to avoid the switching to other biologics.

## 2. Results

### 2.1. Clinical Characteristics

In the study we enrolled 40 (Males/Females, 24/16) patients that underwent different treatments as rescue therapies, namely ciclosporin (*n* = 11), MTX (*n* = 15), NB-UVB (*n* = 7) and apremilast (*n* = 7). The median age in our sample was 44.5 [36.8–51.3], with females undergoing combination therapy later than males (47.5 [38.0–51.3] vs. 42.5 [36.8–51.8], *p* < 0.05). All patients treated with apremilast plus secukinumab and 2 treated with MTX plus secukinumab had PsA. Remarkably, 20 patients had a family history of PsO and/or PsA and 15 were bio-naïve before secukinumab. The burden of comorbidities included systolic hypertension (*n* = 12, 30.0%), chronic obstructive pulmonary disease (COPD) (*n* = 5, 12.5%), diabetes mellitus (*n* = 3, 7.5%) ad uveitis (*n* = 1, 2.5%). For further details see Table 1.

### 2.2. Therapeutic Outcomes

Secukinumab started in monotherapy in all patients included in this study at T0. At baseline, mean PASI was 15.5 [13.0–18.0] and, at week 16, at least PASI 75, with absolute PASI 4 [2.8–4.3], was achieved.

Combination/rescue therapy was administered as follow:

- Apremilast and MTX was prescribed for the entire period of the study, according to in-label dose. The combination therapy with apremilast was reserved to patients that failed a TNF-alpha inhibitor and at least an another inhibitor of the IL-17/23 pathway due to the high pharmaco-economical impact and was approved by the Healthcare Commission of the Institute as compassionate use;
- cyclosporin (dose: 3.5 mg/kg/die) was administered for 3 months, stopped for 1 months and then re-administered for 3 months
- NB-UVB sessions were delivered in 3 cycles of 12 sessions each, starting form a minimum of 0,20 Joule and then increasing up to 1,50 J for each session, according to phototype

Apremilast was privileged in case of occurrent PsA and/or with newly diagnosed enthesitis in patients treated with secukinumab in monotherapy, whilst MTX was preferred in patients with hypertension and PsA. Conversely, NB-UVB and cyclosporine were prescribed in patients without PsA with a prevalent cutaneous flare of PsO; in particular, NB-UVB was preferred in patients with hypertension and cyclosporine in patients that had other concomitant autoimmune or chronic inflammatory diseases (i.e., uveitis).

At T2, when the combination therapy started, patients displayed PASI 8 [7.0–9.0] and a DLQI 13 [12.0–15-0], so a moderate plaque psoriasis with a very large effect on daily life and functionality. After 16 weeks of rescue/combination therapy, they achieved a median absolute PASI (3 [2.8–4.0]) lower than T1 and this positive trend continued also after 52 weeks at T4 (PASI 3[2.0–3.3]). PASI improvement in both T3 and T4 compared to T2 was statistically significant (*p* < 0.05) with Kruskal-Wallis test. Interestingly, no differences in terms of ΔPASI(T4-T2) were found comparing ciclosporin and MTX (*p* = 0.79), ciclosporin and NB-UVB (*p* = 0.78) and MTX and NB-UVB (*p* = 0.66) with Mann Whitney test. No sex differences in ΔPASI(T4-T2) were detected (*p* = 0.92). Conversely, differences were found comparing apremilast to the other combination therapies (ciclosporin (*p* = 0.01), MTX (*p* = 0.005), NB-UVB (*p* = 0.01). These findings justify the clinical preference for apremilast in patients with uncontrolled PsA and a scarce cutaneous involvement. Remarkably, at T4 patients had a DLQI of 3 [2.0–3.3] so a negligible effect of psoriasis on daily life and functionality with a median improvement of 82.8%. All rescue therapy determined a clinical success achieving satisfactory outcomes in terms of PASI and DLQI. None of the patients experienced side effects or switched to other drugs.

**Figure 1:**
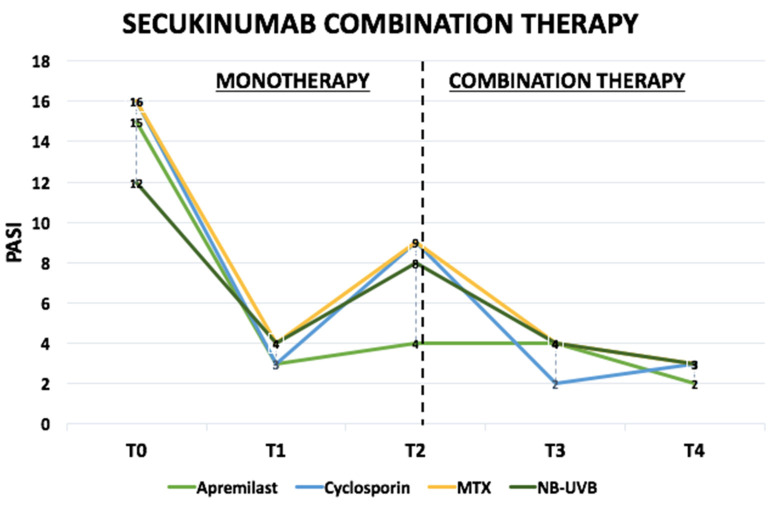
Therapeutic trajectories of psoriatic patients undergoing secukinumab in combination with an another systemic anti-psoriatic drug. MTX: Methotrexate, NB-UVB: Narrow-band UVB, PASI: Psoriasis Area Severity Index. T0: starting secukinumab induction phase, T1: week 16, T2: introduction of the combination/rescue therapy, T3 (16 weeks of combination/rescue therapy), T4 (52 weeks of combination/rescue therapy).

Therapeutic trajectories are visualized in Figure 1 divided for combination/rescue therapies and report PASI values during the analyzed period.

## 3. Discussion

The combination/rescue therapy in all enrolled subjects that experienced a secondary secukinumab failure was safe and contributed to re-achieve and maintain at least PASI 75 without switching to other biologics. Since psoriasis is chronic inflammatory disease characterized by a relapsing-remitting behavior, the final goal of long-term remission remains challenging and flares are possible [19,20]. Despite psoriasis studies on pathogenesis had clarified several aspects, from a therapeutic point of view [39,40,41], anti-psoriatic biological drugs antagonize TNF-α, IL-17/IL-23 pathway and, recently, also JAKs. Thus, interclass switching is a limited option and intraclass switching increases the failure rate configure the actual scenario of progressive implementation of multifailure patients [42,43]. Furthermore, biosimilars are different drugs from the originator but a failure with the originator may implies a potential failure also with the related biosimilar [44,45].

A rationale for switching from TNF-α to IL-17/IL-23 pathway and *viceversa* was demonstrated both in vivo and vitro by Zaba et colleagues [46], whilst a few information are present on JAKS inhibitors. At the same time, no predictive biomarkers are validated for biological therapy in psoriatic patients, excepted the HLA-C*06:02 for ustekinumab [47]. Consequently, in this intricate scenario monotherapy with biologics become more and more risky and some rescue therapies in case of biologic loss of function should be considered and performed, but data are not solid, discordant and/or evaluate a short follow-up (less than 52 weeks) [48].

Among the suggested therapeutic strategies, the European S3-guidelines on systemic treatment of psoriasis vulgaris included the sequential therapy (after the failure of a biologic you stop it and proceed with an another one) [49], the rotational therapy (rotation of two drugs for a fixed amount of time to minimize drug related side effects (i.e., cyclosporine and MTX)) [50] and the combination therapy (simultaneous use of more than one drug) [51]. In particular, combination therapy could be differently performed:

Imbrication: the new biologic is introduced together with an another anti-psoriatic agent that is faster or more focused on a precise symptom and when the biologic starts to be efficient the agent is rapidly dropped;

Cyclic: the biologic loses efficacy and an another anti-psoriatic treatment is performed cyclically to maintain the disease control (i.e., NB-UVB and secukinumab);

Permanent: the biologic loses efficacy and an another anti-psoriatic treatment is synergically performed (i.e., apremilast and secukinumab).

In literature, almost no studies had evaluated the long-term efficacy and safety profile of secukinumab in cyclic or permanent combination therapy to rescue the secondary biologic failure. Thus, the present study offered some insights in term of efficacy and safety to apply this rescue therapy in the daily clinical practice, where the switch to another biologic is also burdened by to the cost of the induction phase.

Furthermore, secukinumab displays an excellent profile of safety and efficacy but 88.9%, 68.5% and 43.8% at 1 year maintains respectively PASI75, PASI90 and PASI100, as shown in the SCULPTURE Extensione Study. Interestingly, real-life data are more encouraging with 82.2% maintained PASI75, 75.3% PASI 90 and 64.4% PASI100 [52]. At the moment the pathogenetic mechanism of secukinumab-related loss of response remains unknown and several hypothesis are present in literature: anti-drug antibodies [53], increased production of TNF-α [46], changes in lifestyle [54,55,56,57], lack of compliance [58] and comorbidities [59]. Thus, different combination therapies may help to counteract the intricate and partially unknown mechanisms of secukinumab secondary failure.

Nowadays, MTX is regarded as the first line of combination therapy for its adjuvant potential and preventive effect against immunogenicity mainly described in TNF-α inhibitors [60]. Despite its large diffusion in the real-life setting, no protocols are validated [61]. We used MTX in case of secukinumab secondary failure in patients with a flare or *de novo* PsA and with hypertension. The other option for PsA, especially in case of new onset enthesitis was apremilast, it acts on the PDE-4 and resets both immune and endocrinological systems [62,63,64]. Conversely, cyclosporine and NB-UVB were destined to patients that experienced cutaneous flares; cyclosporine was privileged in case of severe cutaneous flares with erythema and logistic/physical difficulties to attend the NB-UVB appointments, whilst NB-UVB in patients with hypertension and electrolytes disorders. In case of psoriasis *cum pustulatione* or concomitant new onset pustular psoriasis the logical option will be to combine acitretin, but in the present study we did not face this case.

These options enrich the dermatological *armamentarium* to treat plaque psoriasis, but actually, beside the PASI75 failure at week 16 and drug-related major adverse events, no clear switching criteria are validated, making study comparison very challenging, since switching criteria are usually not even reported. In Italy, that has a universalistic healthcare system, we recently experienced three type of biologic switching: clinically driven (loss of function of the previous drug), pharmaco-economic driven (only from originator to biosimilar or from biosimilar to biosimilar) and COVID-19 pandemic driven (to drugs that deserves less administration per year to avoid hospitals) [21]. Furthermore, comparing universalistic (i.e., Italy) and non-universalistic healthcare systems (i.e., US), two main differences in switching appear: in non-universalistic systems the switching may be performed also in case of insurance changes and the use of biosimilar is extremely rare. Due to these differences the switching literature is not perfectly comparable between the two healthcare systems and real-life data on combination therapy are necessary to improve the daily practice and decrease the incidence of multifailure patients.

Beside the novelty of this study, it presents also some limitations such as the limited sample, the lack of evaluation of lifestyles, family contest and caregivers reported outcomes. Thus, for the limited sample we were not able to synergically evaluate all this social variables that seriously modulate patients’ compliance to the therapy. Furthermore, results referring to the subgroups of combined therapies need to be validated in larger, dedicated studies. In particular, we reserved apremilast only to psoriatic patients treated with secukinumab with an uncontrolled PsA and a mild residual PASI for pharmaco-economical reasons.

## 4. Materials and Methods

### 4.1. Study Design

This multicenter retrospective observational study involved three Italian primary referral centers (IRCCS Istituto Ortopedico Galeazzi (Milan), IRCCS San Donato Hospital (San Donato, Milan) and IRCCS San Gallicano Hospital (Rome)) and was performed from February 2016 to December 2020. All psoriatic patients undergoing Secukinumab that experienced a secondary loss of function after >1 year and were treated with an additional systemic therapy were consecutively enrolled. Patients follow-up started at T0: starting secukinumab monotherapy induction phase, T1: week 16 of secukinumab monotherapy, T2: introduction of the rescue/combination therapy, T3: 16 weeks after starting combination therapy, T4: 52 weeks after starting combination therapy.

The study was conducted according to the guidelines of the Declaration of Helsinki, and approved by the Institutional Review Board of San Raphael Hospital (protocol code: 178/INT/2021 and date of approval: 10 November 2021).

### 4.2. Inclusion and Exclusion Criteria

Patients with (a) plaque psoriasis for more than 6 months duration, (b) treated in label with secukinumab (c) experiencing a secondary loss of efficacy after 52 weeks of secukinuma monotherapy, (d) with high adherence to the therapies (Brief Adherence Rating Scale (BARS) > 90%) were enrolled [65,66].

Conversely, patients were excluded in case of (a) type of psoriasis different from or concomitant with plaque psoriasis (i.e., pustular psoriasis), (b) undergoing fasting of particular diet regimens (i.e., intermitten circadian fasting), (c) addictions (i.e., drugs [67] or alcohol abuse (Alcohol Use Disorders Identification Test (AUDIT > 7)) [68], (d) chronic infectious diseases (i.e., tuberculosis, HIV, hepatitis B and C) [14,15,49], e) use of drugs capable to trigger plaque psoriasis flares [68,69,70], f) patients with persistent itch during monotherapy (persistent itch Visual Analogue Scale (VAS) > 4) [71,72,73].

### 4.3. Clinical Evaluation

All the enrolled patients were assessed by two expert (>5 years experience) board certified dermatologists (G.D. and P.D.M.P. at Galeazzi Hospital, P.M. and G.D. at San Donato Hospital, A.P. and A.M. at San Gallicano Hospital) at T0, T1, T2, T3 and T4. Demographics and clinical information were carefully collected. Each visit, these above clinical scales were measured: Psoriasis Area Severity Index (PASI) [74], Psoriasis Epidemiology Screening Tool (PEST) [75], itch-VAS [76] and Dermatology Life Quality Index (DLQI) [77]. The loss of efficacy in T2 was defined as a PASI increase greater than 20% or an increased absolute PASI greater than 3 in two consecutive assessments.

Combination therapies with the systemic drug were prescribed in line with the “Italian guidelines on the systemic treatments of moderate-to-severe plaque psoriasis” in terms of dosage and frequency [78], whilst the NB-UVB was used following the “Tuscan consensus on the use of UVBnb phototherapy” [79] and the principles of diet changes in minimal erythema dose [80] and photoadaptation [81].

### 4.4. Statistical Analysis

Preliminarily data were evaluated to detect potential outliers and then normality was verified with D’Agostino and Pearson omnibus normality test. Variables normally distributed were reported as means ± standard deviation, conversely as median and interquartile range. Categorical parameters were reported as percentages. Statistical analyses were performed with SPSS version 24.0 for Windows (IBM, Armonk, NY, USA). The difference between data obtained at T2 and T4 was calculated separately for each therapeutical group, and the different groups were then compared with Kruskal Wallis test, followed by Mann Whitney test for assessing differences between groups. Comparison between male and female data was also performed using Mann Whitney test. Statistical significance was established with *p*-value < 0.05.

## 5. Conclusions

Secukinumab remains a safety and effective drug for PsO patients also in the IL-23 and JAK inhibitors era. The rescue therapy is a valid therapeutic option in case of secukinumab secondary failure to avoid switching and to preserve newest drugs for future emergency or uncontrolled flares.

Multifailure patients constantly grow and dermatologists are facing this challenge with a limited number of therapeutically interesting pathway to block; in this intricate scenario the conscious use of combination therapy is a valid and relatively inexpensive strategy to treat PsO patients. Further studies should be performed to establish precise protocols of combination therapy and switching criteria.

## Figures and Tables

**Table 1:** Clinical and therapeutic characteristics of the enrolled patients stratified for the combination therapy type.

	Secukinumab + Apremilast (*n* = 7)	Secukinumab + Cyclosporin (*n* = 11)	Secukinumab + Methotrexate (*n* = 15)	Secukinumab + NB-UVB (*n* = 7)
Age (median [IQR], years old)	42.0 [37.0–47.5]	42.0 [33.5–51.5]	48.0 [38.5–50.0]	49.0 [41.0–53,5]
Gender, M/F (*n*)	5/2	7/4	9/6	3/4
Family history of psoriasis (*n* (%))	4 (57.1)	7 (63.6)	6 (40.0)	3 (42.9)
Psoriatic Arthritis (*n* (%))	7 (100.0)	0 (0.0)	2 (13.3)	0 (0.0)
Comorbidities (*n*, (%))				
- Hypertension	2 (28.6)	0 (0.0)	4 (26.7)	6 (85.7)
- COPD	0 (0.0)	2 (18.2)	0 (0.0)	3 (42.9)
- Diabetes	0 (0.0)	0 (0.0)	2 (13.3)	1 (14.3)
- Uveitis	0 (0.0)	1 (9.1)	0 (0.0)	0 (0.0)
Biologics naive (*n* (%))	3 (42.9)	5 (45.5)	7 (46.7)	4 (57.1)
Secukinumab monotherapy longevity (months, median [IQR])	9 [8.5–10.5]	9 [7.5–11.0]	11 [9.5–13.0]	12 [10.0–13.0]
Combination therapy duration, (months, median [IQR])	12 months	3+ 3 months	12 months	3 cycles of 12 phototherapy sessions each
PASI (median [IQR])				
➣ T0	15 [14.0–17.0]	16 [15.0–20.5]	16 [13.5–18.0]	12 [12.0–13.5]
➣ T1	3 [2.0–4.0]	3 [3.0–4.0]	4 [3.0–5.0]	4 [3.0–4.5]
➣ T2	4 [2.5–5.0]	9 [8.0–9.5]	9 [8.0–10.0]	8 [7.5–9.0]
➣ T3	4 [2.5–4.0]	2 [2.0–3.0]	4 [3.0–5.0]	4 [3.0–4.0]
➣ T4	2 [0.5–2.0]	3 [2.0–3.5]	3 [3.0–3.5]	3 [2.0–4.0]
DLQI (median [IQR])				
➣ T2	16 [14.0–17.0]	14 [13.0–15.0]	12 [11.5–13.5]	12 [10.5–12.5]
➣ T4	2 [2.0–3.0]	2 [1.5–3.0]	3 [2.0–3.5]	3 [2.5–4.5]
PASI 75 (*n*, (%))				
➣ T3	5 (71.4)	8 (72.7)	8 (53.3)	3 (42.9)
➣ T4	4 (57.1)	9 (81.8)	13 (86.7)	4 (57.1)
PASI 90 (*n*, (%))				
➣ T3	1 (14.3)	2 (18.2)	1 (6.7)	0 (0.0)
➣ T4	1 (14.3)	0 (0.0)	0 (0.0)	0 (0.0)
PASI 100 (*n*, (%))				
➣ T3	0 (0.0)	0 (0.0)	0 (0.0)	0 (0.0)
➣ T4	2 (28.6)	0 (0.0)	0 (0.0)	0 (0.0)
Side-effects (*n*, (%))	0 (0.0)	0 (0.0)	0 (0.0)	0 (0.0)

COPD: Chronic obstructive pulmonary disease, DLQI: Dermatological life quality index, F: Female, IQR: Interquartile range, M: Male, PASI: Psoriasis Area Severity Index.

## Data Availability

The data presented in this study are available on request from the corresponding author (dr.giovanni.damiani@gmail.com). The data are not publicly available due to privacy issues.
